# Taking Particle Tracking into Practice by Novel Software and Screening Approach: Case-Study of Oral Lipid Nanocarriers

**DOI:** 10.3390/pharmaceutics13030370

**Published:** 2021-03-10

**Authors:** María Plaza-Oliver, Emilio L. Cano, María Mar Arroyo-Jimenez, Matías Gámez, María Victoria Lozano-López, Manuel J. Santander-Ortega

**Affiliations:** 1 Cellular Neurobiology and Molecular Chemistry of the Central Nervous System Group, Faculty of Pharmacy, University of Castilla-La Mancha (UCLM), 02071 Albacete, Spain; maria.plaza@uclm.es (M.P.-O.); Mariamar.Arroyo@uclm.es (M.M.A.-J.); mvictoria.lozano@uclm.es (M.V.L.-L.); 2 Regional Centre of Biomedical Research (CRIB), University of Castilla-La Mancha (UCLM), 02008 Albacete, Spain; 3 Quantitative Methods and Socio-economic Development Group, Institute for Regional Development (IDR), University of Castilla-La Mancha (UCLM), 02006 Albacete, Spain; Matias.Gamez@uclm.es; 4 Data Science laboratory, Rey Juan Carlos University, 28933 Madrid, Spain

**Keywords:** particle tracking, diffusion, oral lipid nanocarriers, data processing, R software, screening of trajectories

## Abstract

The success on the design of new oral nanocarriers greatly depends on the identification of the best physicochemical properties that would allow their diffusion across the mucus layer that protects the intestinal epithelium. In this context, particle tracking (PT) has arisen in the pharmaceutical field as an excellent tool to evaluate the diffusion of individual particles across the intestinal mucus. In PT, the trajectories of individual particles are characterized by the mean square displacement (*MSD*), which is used to calculate the coefficient of diffusion (*D*) and the anomalous diffusion parameter (*α*) as $MSD=4D\tau^{\alpha }$. Unfortunately, there is no stablished criteria to evaluate the goodness-of-fit of the experimental data to the mathematical model. This work shows that the commonly used *R*^2^ parameter may lead to an overestimation of the diffusion capacity of oral nanocarriers. We propose a screening approach based on a combination of *R*^2^ with further statistical parameters. We have analyzed the effect of this approach to study the intestinal mucodiffusion of lipid oral nanocarriers, compared to the conventional screening approach. Last, we have developed software able to perform the whole PT analysis in a time-saving, user-friendly, and rational fashion.

## 1. Introduction

Lipid nanocarriers have shown a great potential as oral drug delivery systems [1,2]. These nanocarriers can be formulated either as digestible or non-digestible, depending on the cargo molecule and the target of the formulation. In any case, there are components of the gastrointestinal tract such as electrolytes and macromolecules that may modify the outer composition of the lipid droplets [3,4]. This may result in a heterogeneous-shell system that must overcome the mucus barrier to reach its target, i.e., the intestinal epithelium. Under this scenario, it is mandatory to work with experimental tools that properly analyze the mucodiffusion capacity of each individual particle. Those particles would be the ones that reach the intestinal epithelial cells, an additional biological barrier for the local treatment or for achieving systemic drug levels [5].

Particle tracking (PT) is a powerful microscopy technique that enables the quantification of single particle diffusion in non-diluted and complex biological media [6,7,8,9,10]. The use of PT in oral drug delivery determines the diffusion of nanocarriers across the intestinal mucus and assesses those factors like size, hydrophobicity, or charge that affect mucodiffusion [3,11,12,13]. Consequently, PT may help to understand the biological behavior of oral nanocarriers and be a useful tool for researchers in the rational design and fine tune-up of these formulations. In brief, PT experiments uses fluorescence microscopy with high-sensitivity cameras to record time-lapse videos of fluorescently-labeled nanocarriers incubated with intestinal mucus or mucin reconstituted gels. Then, each particle is located throughout the different frames of the videos and these positions are linked to obtain plausible trajectories. A deeper vision on how to perform these experiments and further technical details are excellently reviewed elsewhere [6,9,10].

One of the main strengths of PT relies on its capacity to track individual particles. This makes it feasible to identify and characterize heterogeneity in the behavior of the different particles that constitute the sample, which is particularly relevant when designing novel nanocarriers [5,14,15]. The oil/water interface composition of each particle will determine its individual interaction with the intestinal mucus, being possible to classify them into the different populations that form a batch [3,6,9,10]. This is especially relevant in pharmaceutical technology as it allows a more accurate estimation of the fraction of the oral administered dose that could reach the intestinal epithelium at a certain time point.

Individual trajectories are characterized by the mean square displacement (*MSD*), given the *x* and *y* position data of each particle. In viscous fluids, such as intestinal mucus, *MSD* is proportional to the coefficient of diffusion (*D*). A typical situation that happens in biological media is when particles encounter obstacles that hamper their free diffusion, i.e., intestinal mucus, then their movements suffer a deviation from free Brownian motion. This process is called anomalous diffusion and can be represented by the dimensionless parameter *α* (anomalous diffusion coefficient) [8,9,16,17]. In these cases, the two-dimensional diffusion *MSD* can be calculated using the following Equation (1) [6,8,14,16,18]:

(1) $$MSD=4D\tau^{\alpha },$$

where *τ* is the lag time. This fitting is applied to every single particle, generally thousands of them within a sample. In this sense, it is mandatory to follow or develop a decision-making tool to evaluate the goodness-of-fit of the raw experimental data to the theoretical model. Currently, there is a lack of well-established protocols to filter and analyze the trajectories as well as of information about the data processing criteria followed in the specialized literature [19]. Within this scenario, the coefficient of determination of each particle (*R*^2^) is commonly used as screening parameter, dismissing the particles for which the $R^{2}$ from Equation (1) fitting is lower than certain threshold (0.9, 0.8, 0.7, etc., …) [20,21,22,23,24]. From a mathematical point of view (Equation (2), *R*^2^ is expressed as:

(2) $$R^{2}=1-\frac{RSS}{TSS},$$

where *RSS* is the residuals sum of squares and *TSS* is the total sum of squares. The term $R^{2}$ is probably the most accepted expression to determine the goodness-of-fit in statistical models. However, it is important to highlight that for close to horizontal lines, that is when the *MSD* of a particle presents a minimal dependence on *τ*, RSS/TSS→1. Under this scenario, even if the regression analysis of the *MSD* vs. *τ* experimental data with respect to the theoretical model shows a good fitting, $R^{2}\to 0$. This mathematical issue is especially relevant for the study of the interaction of nanocarriers with the intestinal mucus.

**Figure 1:**
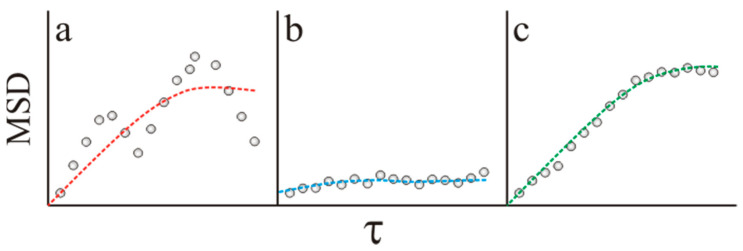
Three possible scenarios in the regression analysis of experimental Mean Square Displacement (*MSD*) vs. lag time (*τ*) data (dots) to the $MSD=4D\tau^{\alpha }$ theoretical model (dashed line). (**a**) Erratic trajectories due to uncontrollable experimental artifacts ($MSD=MSD\left(\tau \right);\;R^{2}\to 0$). (**b**) Nanoparticles unable to diffuse across the intestinal mucus (MSD≠MSD(τ); $R^{2}\to 0$). (**c**) Nanoparticles able to diffuse across the intestinal mucus ($MSD=MSD\left(\tau \right);\;R^{2}\to 1$).

The study of the mucodiffusion capacity of nanocarriers can bring three main scenarios, illustrated in Figure 1. Bearing this mind, using $R^{2}$ as the unique screening parameter of raw *MSD* vs. *τ* set of data may result in the consideration of those particles with a limited capacity to overcome the intestinal mucus as experimental artifacts or erratic trajectories (as $R^{2}\to 0$). It is important to highlight that the exclusion of this non-diffusing population of particles from the final data analysis may result in the overestimation of the therapeutic capacity of a nanocarrier. This inaccurate screening issue would lead to the following chain reaction:

- Underestimation of the fraction of the nanocarrier retained in the mucus layer.
- Overestimation of the potential therapeutic effect of the nanocarrier after their oral administration.
- Difficulties in stablishing a clear relationship between the physicochemical properties of the nanocarrier and its capacity to overcome the intestinal mucus.
- Amplified in vitro–in vivo divergence.
- Misuse of test animals in the subsequent in vivo assays.

This work aims to determine the reliability of using *R*^2^ as the unique statistic parameter to determine the goodness-of-fit of the *MSD* vs. *τ* curves obtained in PT experiments. Then, we have evaluated the limitations of this conventional approach using model polystyrene nanoparticles as control (PS_NPs_) as well as oral lipid nanocarriers (case study). The acquired knowledge is gathered into a free and user-friendly software for PT analysis that aims to contribute to the rational design and development of nanocarriers in drug delivery.

## 2. Materials and Methods

### 2.1. Polystyrene Nanoparticles

Fluorescent carboxylic polystyrene nanoparticles (PS_NPs_) (λex = 576 nm, λem = 596 nm) were formulated and characterized by Ikerlat S.A. Laboratories (Gipuzkoa, Spain). Their size and polydispersity index were 123 ± 1 nm and 1.008, respectively. Plain PS_NPs_ were used as mucoadhesive control [25,26]. The same PS_NPs_ coated with Pluronic^®^ F127 (PF127) were used as mucodiffusive model nanoparticles [6,11,25,27].

### 2.2. Formulation of Lipid Nanocarriers (Case-Study)

A formulation of o/w nanoemulsions was selected as representative lipid nanocarriers and obtained using the solvent displacement technique as recently described [3]. The organic phase, constituted by 5 mL of acetone, 38 mg of α-tocopherol (Sigma, Madrid, Spain) and 17 mg of ascorbyl-dipalmitate (CombiBlocks, San Diego, CA, USA) was added to 10 mL of ultrapure water. Then, solvents were rota-evaporated to yield a final volume of 5 mL. The obtaining of partially digested nanoemulsions was achieved by incubating the particles with a mixture containing pancreatin, porcine bile salts (5 mg/mL), and CaCl_2_·2H_2_O (0.3 mg/mL) (Sigma, Spain) diluted in simulated intestinal fluid (USP XXIX guidelines) for 24 h at 37 °C [3,28]. Partially digested nanoemulsions were recovered after centrifugation at 5000 rpm for 10 min.

### 2.3. Intestinal Mucus Extraction

Porcine intestinal mucus is a well stablished model of human intestinal mucus [3,11,12,29]. For that purpose, adult-porcine small intestines were obtained from a local slaughterhouse and washed with 2 mM phosphate-buffer saline (PBS) The intestinal mucus was scraped from the intestine. Finally, 1–2 mL aliquots of mucus were obtained and stored at −20 °C until their use.

### 2.4. PT Videos Recording and Data Collection

Mucoadhesive or mucodiffusive PS_NPs_ were diluted in 2 mM PBS. Then, particles and mucus samples were heated up to 37 °C to simulate the body temperature. After that, 10 µL of the diluted PS_NPs_ were gently mixed with 100 µL of mucus, and 10–15 µL of this sample was placed on a microscope slide with a double-adhesive sticker (120 µm thickness) (Sigma, Portland, OR, USA) before being covered with a cover glass. The PT experiments were performed using a Nikon microscope with a PLAN APO 100 × 1.4 oil-immersion objective placed on an anti-vibration table (Vision station, Newport). The microscope was equipped with a thermostatic platen (Tokai hit) to maintain the samples at 37 °C during the experiments. The Brownian motion of the particles was recorded with an Andor Zyla 4.2 camera (pixel resolution of 70 nm). All the movies were recorded at a minimum focal plane of 8–12 µm from the cover glass to avoid erratic trajectories affected by the interaction of the P-NPs with the cover slip [30]. For each sample, we recorded 20 movies at 100 fps, obtaining a minimum of 100 trajectories per movie.

### 2.5. PT Data Processing

Nd2 Nikon video files were analyzed with the tracking module of the NIS elements software (Nikon Corp., Melville, NY, USA). This software correlates the position of a particle along the sequence of frames with the algorithm developed by Jaqaman et al. [31]. Under the linear assignment problem (LAP) mathematical framework [32], this algorithm faces some of the inherent problems of PT, such as particle concentration, motion heterogeneity, particles merging/splitting trajectories or particles disappearance from the focal plane. Standard deviation multiplication factor was set to 2.

This approximation to the multiple-hypothesis tracking (MHT), one of the most accurate solutions to PT [33], was used for the determination of the evolution of the *MSD_ijk_* as a function of the time in each *i* movie (*i* = 1, …, 20) for each one of the *j* trajectories (*j =* 1, … *m_i_*, where *m_i_* is the number of trajectories in video *i*); *k* is the segment id (*k* = 1, …, *n_ij_*, being *n_ij_* the number of segments in trajectory *j* in video *i*). To minimize experimental artifacts, data processing only considered those trajectories with at least 10 segments (*n_ij_* ≥ 10) [34].

### 2.6. Analysis of the Trajectories

Once MSD vs. lag time curve was trimmed at the selected lag time (up to 1 s, detailed information is described in Section 3.1), it was necessary to determine the goodness-of-fit for each trajectory to the mathematical model, not considering those with a poor fitting for the final *D* and *α* estimation. First, we estimate *D* and *α*, i.e., ${\hat{D}}_{ij}$ and ${\hat{\alpha }}_{ij}$, respectively, for each particle. We use the two main methods for estimating the model parameters (Equation (3), namely: (1) a non-linear fit of Equation (1), for which several algorithms are available; and (2) a linear fit of the log transformation of Equation (1):

(3) log(MSD)=log(4D)+α·log(τ)+ε.

In the former approach, we directly get estimations and confidence intervals for the parameters *D* and *α*. In the latter, least squares estimates are obtained for the regression line in Equation (3), i.e., $y=\beta_{0}+\beta_{1}x+\varepsilon$. The estimated slope of a given particle, ${\hat{\beta }}_{1}$, is directly ${\hat{\alpha }}_{ij}$, the estimate of the *α* parameter, whereas ${\hat{D}}_{ij}$ must be computed through the estimated intercept, ${\hat{\beta }}_{0}$, as follows (Equation (4):

(4) $${\hat{D}}_{ij}=\frac{e^{{\hat{\beta }}_{0}}}{4}.$$

Then, for each fitted model, i.e., particle trajectory, and for both methods (non-linear, and linear transformation), different error measurements can be computed, including the coefficient of determination, *R*^2^. In the PT screening, it is common to select the trajectories whose linear model *R*^2^ surpasses a given threshold, say 0.9, or any other criteria selected by the researcher [20,21,22,23,24]. This may result in misleading decisions, as discussed previously and in Section 3. Our approach is to combine further error measures, compare between fitting methods, and use combined thresholds to get a more realistic awareness of the particle diffusion behavior.

#### 2.6.1. Software Implementation

It is frequent that PT analysts invest much time arranging the results from diverse scientific software into spreadsheets, as well as manually screening the trajectories and figuring out the diffusion properties of their samples. Despite there being some open-source software available for PT users, none of them includes the combination approach for the screening and assessment of the goodness-of-fit that is proposed in this work. In this sense, we have developed a PT software that implements our methodology, optimizing the analysis while keeping the reliability of the results.

For the development of PT software, we relied on the most recent version of the R Statistical Software and Programming Language (currently R 4.0.2) (Vienna, Austria) [35]. The R scripts are implemented in a Shiny application [36], which are web applications that run locally or in a web server, and allow user interactivity, reactive programming, and responsive design. A detailed overview of the application and its potential is included in Section 3.4.

#### 2.6.2. Modular Implementation

We have developed a dedicated module to get a better insight on the interaction of nanocarriers with the intestinal mucus barrier using the advantages offered by R scripts and Shiny applications. This module gives quantitative information about different key parameters used to understand the interaction of each individual particle of a formulation with the mucus [13,26,37]:

- $D_{m}/D_{r}$**:** Although some authors use raw D in mucus (*D_m_*), derived from MSD analysis, for the evaluation of the mobility of particles, others tend to compare *D_m_* of the sample with a reference (*D_r_*). More concretely, it is common to express *D_m_* compared to *D* measured in a simple reference media, such as saline (*D_saline_*) or water (*D_w_*). In this way, *D_m_/D_w_* is calculated and particles are considered diffusive at *D_m_/D_w_* ~ 1 [10,13]. As an alternative, *D_m_* might also be compared to *D_m_* of a mucodiffusive control (*D_m control_*), obtaining *D_m_/D_m control_* value. The *D_m_/D_m control_* ratio gains relevance when different samples of intestinal mucus are used to perform a PT experiment, since it enables to correct the variability occuring due to the intra- and inter-heterogeneity of the mucus porcine samples, e.g., different viscosity [3,38,39].
- $D_{long}/D_{short}$**:** An interesting option to determine whether the particles are able or not to diffuse across the mucus is to determine the diffusivity factor for each individual particle ($DF=D_{long}/D_{short}$) [3,14,40]. As for *D_m_/D_r_*, it is possible to calculate *D* for the same trajectory at two different temporal scales, namely the lag time threshold and an additional shorter lag time (e.g., 1 and 0.2 s); referring $\;D_{long}$ to D. Free diffusing particles may display a similar diffusion pattern at long (1 s) and short (0.2 s) lag times, i.e., *DF* ≥ 0.9. However, those particles that interact with the mucus display an *MSD* vs. *τ* curve which slopes decrease with the time, that is *DF* < 0.9 [11,14,25].
- ***α*:** As commented above, while free-diffusing particles display an *α* ≈ 1, mucus-retained particles present *α* < 1. Depending on the mucins-particle strength of interaction, the transport mode of each individual particle can be sorted as follows: (i) immobile (*α* < 0.2); (ii) hindered (0.2 < *α* < 0.4); (iii) subdiffusive (0.4 < *α* < 0.9); and (iv) diffusive (0.9 < *α*) [3,11,13,37,41]. On the other hand, *α* > 1 is usually associated with super-diffusive particles. This may occur in case the sample is not properly sealed, which may lead to flow channels through which particles rapidly diffuse. Thus, *α* values > 1.1 are normally discarded from the results. Similarly, the fitting of equations Equation (1) or Equation (3) may result on an estimation of $\hat{\alpha }<0$. In these cases, $\hat{\alpha }$ is considered to be equal to 0.

#### 2.6.3. Mean and Median Paradigm in Real Samples

To achieve an accurate interpretation of the obtained results, it is highly advisable to rationally select which statistical measurement fits best to express them. In this sense, the most used parameters are the mean and the median. The mean might be an appropriate estimator when data follow a Gaussian distribution, but in conditions of high heterogeneity with extreme behaviors, the use of the mean might lead to misleading results. In these situations, selecting the median may be a more suitable choice, since it would be more representative than the mean, and subsequently less biased [42]. This could be the situation of lipid nanocarriers that are in the gastrointestinal tract, exposed to enzymes that may lead to heterogeneous mixtures including non-digested, partially-digested, and totally digested particles [43,44]. Each of these populations may interact differently with the intestinal mucus (see case-study section). For this reason, it is useful to perform some exploratory data analysis of the distribution of the estimators, the *R*^2^ and the error measured using histograms and numeric summaries. Apart from helping the PT user to select the more suitable statistic, this analysis may contribute to the quick understanding of the heterogeneity of the sample. This is especially relevant when only a small fraction of the sample is required to achieve the desired therapeutic effect. However, this information could be missed in case of systematically working only with the mean of the results.

## 3. Results and Discussion

It is undoubted that PT is a technique of which fundaments are well stablished and therefore, highly promising for drug delivery. Nevertheless, there is not a generally accepted method for data analysis, so researchers may encounter difficulties like the generation of a large amount of data or the screening of the raw data to determine the goodness-of-fit of the raw trajectories to the mathematical model. This is internally solved by researchers but this information is usually not available, so many questions regarding PT data analysis remain unanswered. This work aims to contribute to PT data analysis by providing a novel screening approach for particle trajectories, which has been additionally applied to lipid nanocarriers, a well-known system in drug delivery. Finally, this information has been gathered into a publicly available software to enable the robust analysis of PT data.

### 3.1. Video Recording and Data Processing Parameters

The pixel resolution of the microscope setup, described in Section 2.4, was 70 or 35 nm (when 2× magnification lens were placed between the microscope and the CCD camera), which was small enough to have a good resolution of the nanoparticles movement and also meet the Nyquist sampling theorem [6,10]. Additionally, this pixel size was large enough to collect the adequate number of photons per pixel during the image acquisition time (10 ms) with a good signal/noise (S/N) ratio. Longer image acquisition time might improve the S/N ratio but could also lead to underestimation of the diffusivity of the particles.

Standard deviation multiplication factor was set to 2. Lower values reduced the localization perimeter of a particle from frame to frame, which dramatically decreased the number of available trajectories per movie (data not shown). Increasing the standard deviation multiplication factor, extended the searching perimeter from frame to frame which may result in experimental artifacts by merging different trajectories.

**Figure 2:**
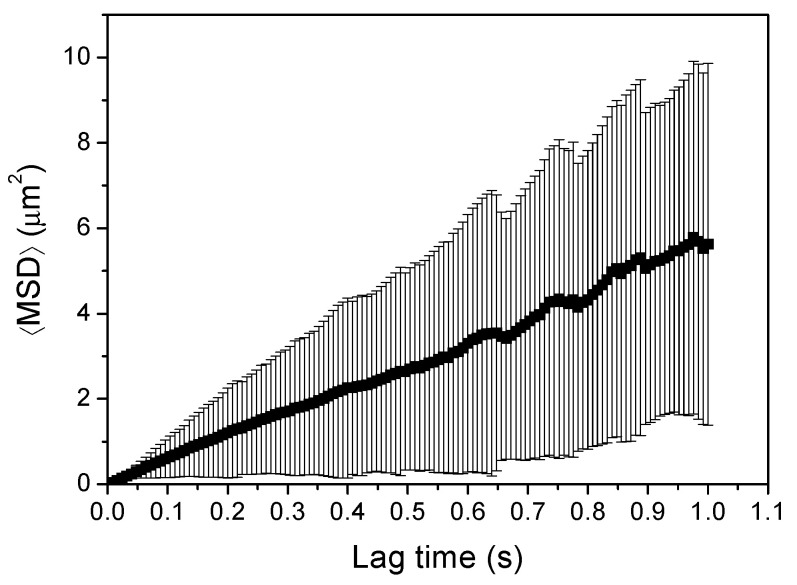
MSD estimation as a function of lag time. The statistical error of the estimation (vertical bars) increases as lag time rises.

The effect that lag time has on the accuracy of PT data analysis has been previously described [10,16,45,46]. In this sense, it is widely accepted that increasing lag times enhances uncertainty and statistical errors of the calculated MSD [10,46], as there are less trajectories at longer lag times and therefore the calculation of MSD would include less statistically significant trajectories (see Figure 2). On this basis, Zagato et al. recommend including 25% of lag times for a robust MSD analysis [10]. Kepten et al. suggested shortening lag time in term of the magnitude of the measurement error. For a medium measurement error, they recommended taking lag times 10–20% of the length of trajectories in the case of short trajectories, and 4–7% in the case of longer trajectories [16]. On the other side, selecting too short lag times could contribute to the overestimation of the diffusion, since particles tend to stabilize or stop their diffusion over time, and thus their true MSD is smaller than the MSD they show at short lag times. Additionally, static (noise) PT errors are more pronounced at shorter lag times [6].

Despite these approximations, there is not an optimal lag time associated with the best estimation of MSD [16,45]. In line with the literature, the model particles PS_NPs_ had an overestimation of their D values in saline at short lag times (0.3 s), whereas the increase of the lag time up to 1 s led to similar D to those obtained by DLS (data not shown). Therefore, the following experiments were performed at a maximum value of 1 s for the lag time, which is consistent with the lag time threshold commonly used by other authors [26,37,47]. In this sense, it is worth to remark that some currently available commercial PT software systematically work with short lag times, i.e., few segments (*n* = 3). Thus, PT users should be cautious and adjust this parameter to ensure reliable MSD calculations.

### 3.2. Analysis of the Trajectories

One of the distinctive features of PT from other techniques is its potential to follow individual trajectories instead of providing information about mean or ensemble diffusion capacity of the whole formulation. Data analysis should initially consider whether raw *MSD* vs. *τ* data fit to the theoretical model, i.e., $MSD=4D\tau^{\alpha }$. This screening is highly relevant but not usually included in the description procedures of PT data analysis. Indeed, it is common to consider as reliable trajectories only those with a goodness of fit higher than a certain threshold of the coefficient of determination (*R*^2^ ≥ 0.9, 0.8, 0.7, …). By doing so, the reproducibility of PT experiments among different laboratories might be compromised; indeed, it can also lead to overestimation of the potential of nanocarriers to pass across a biological barrier, like the intestinal mucus. Thus, this study aims to develop a robust method for the analysis of PT raw data.

It is well-known that a value of *R*^2^ close to 1 ensures a good linear adjustment as it represents the proportion of variability explained by the model. In this line, A. Matysik and R.S. Kraut proposed excluding PT trajectories with *R*^2^ values lower than 0.9. Their filtering approach also included additional criteria, such as the minimum number of frames and a minimum and maximum D and average trajectory intensity [20]. In other recent papers, the *R*^2^ threshold was set at 0.8 [21,22] or even at lower values, such as 0.5 [24].

**Figure 3:**
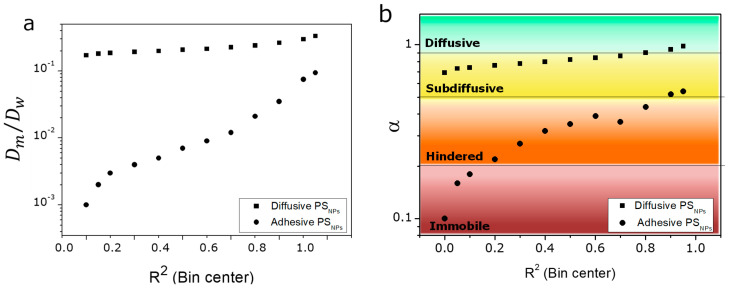
Impact of $R^{2}$ on (**a**) D_m_/D_w_, (**b**) α value of PS_NPs_.

Bearing this in mind we studied the effect of the *R*^2^ trim on the estimated *D* and *α* of both the mucoadhesive and mucodiffusive model PS_NPs_ (expressed as *D_m_*/*D_w_*). Briefly, $D_{m}/D_{w}\to 1$ or α→1 indicates a mucodiffusive behavior, where $D_{m}/D_{w}\to 0$ or α→0 implies that the nanocarriers are stuck on the mucus [3,11,26]. Interestingly, the narrow down of *R*^2^ had a minor effect on both parameters for the mucodiffusive PS_NPs_ (Figure 3), whereas *D_m_*/*D_w_* and *α* parameters of the mucoadhesive counterpart clearly depended on the *R*^2^ threshold. If those trajectories with *R*^2^ below 0.7–0.9 are not considered, we observed an enhancement of both *D_m_*/*D_w_* and *α* values. In fact, *D_m_*/*D_w_* obtained with $R^{2}>0.7–0.9$ criterion was two orders of magnitude higher than the one obtained including trajectories with lower *R*^2^ (Figure 3A). Regarding *α*, the increase of the *R*^2^ threshold shifted from a totally stuck (*α* < 0.2) to a subdiffusive pattern (Figure 3B). PS_NPs_ is a well-known mucoadhesive formulation [47], which does not correlate with the results obtained even at $R^{2}>0.5$.

**Figure 4:**
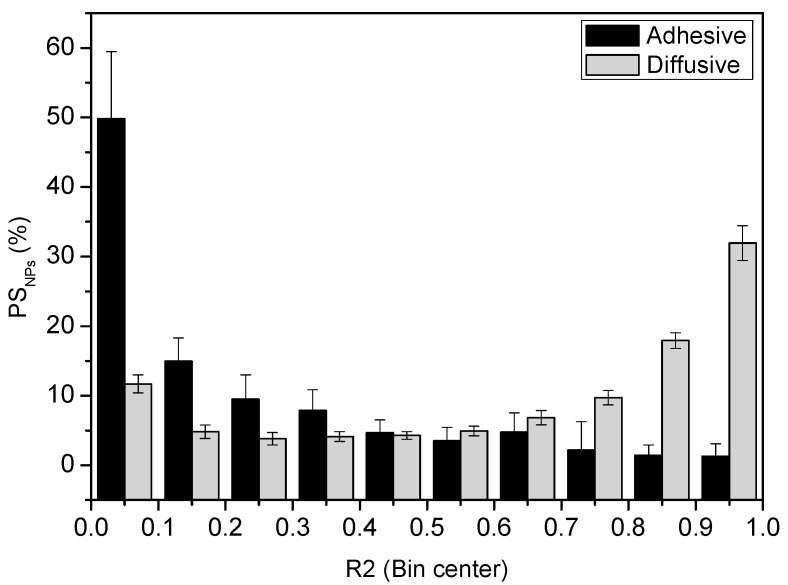
Distribution of trajectories (%) of mucoadhesive (black columns) and mucodiffusive (grey columns) PS_NPs_ as a function of $R^{2}$. Normally, high $R^{2}$ values are associated with diffusive trajectories.

These results can be more easily understood by analyzing the trajectories distribution of each formulation as a function of *R*^2^. The bar chart included in Figure 4 shows the population distribution of the observed trajectories for the mucoadhesive and mucodiffusive PS_NPs_. Nearly 70% of the trajectories from the mucodiffusive PS_NPs_ had $R^{2}\geq 0.6$, which clearly shows that only a small fraction of the mucodiffusive PS_NPs_ will have low *R*^2^ values. This has a reduced impact on *D* or *α* and low *R*^2^ values most probably come from trajectories that do not fit to the theoretical model, e.g., erratic trajectories or experimental artifacts.

Interestingly, ~75% of the trajectories of the mucoadhesive PS_NPs_ presented a $R^{2}\leq 0.3$. A deeper analysis of this set of data showed that only ~10% of the whole formulation have a $R^{2}\geq 0.5$ and just the ~5% a $R^{2}\geq 0.7$. This is in line with the work recently published by A.S Hansen et al. [48]. They also concluded that the MSD fitting with high *R*^2^ values may lead to misleading results. Concretely, they observed that MSD at *R*^2^ ≥ 0.8 only used around 4–5% of the total number of trajectories.

This clearly indicates that using only *R*^2^ as the screening parameter can compromise the validity of the results for low diffusion formulations. Indeed, it is important to highlight that *R*^2^ is excluding not only erratic trajectories, but also those with a good fitting to the mathematical model but low dependence of *MSD* with *τ*. This exclusion of adhesive trajectories and the consequent overestimation of the diffusion could be explained by how *R*^2^ is calculated. Certainly, *R*^2^ is defined as the variation of the *y* axis variable (*MSD*) regarding the *x* axis variable (*τ*). In the case of adhesive particles, *MSD* does not tend to significantly increase along time, so the expected linear fitting is a close to horizontal line and therefore if RSS→TSS, *R*^2^ would be ~0 (see Equation (5) and its corresponding explanation). This is especially relevant when designing novel oral drug nanocarriers, as discriminating non-fitting and mucus-stuck particles is required for not overestimating the potential of the nanocarriers.

### 3.3. Case Study: Analysis of the Mucodiffusion of Oral Lipid Nanocarriers

Lipid nanocarriers are referent in oral drug delivery. These versatile systems can be formulated either as digestible or non-digestible nanocarriers depending on the cargo and the target selected [49]. Once in the intestinal lumen, lipid nanocarriers can be coated by intestinal enzymes, co-enzymes, and bile salts, leading to the formation of an intestinal protein corona I-PC [3,4]. Depending on the specific physicochemical characteristics of the formulation, this enzymatic coating can induce the null, partial, or total digestion of the oil droplets. This process triggers the partition of the formulation in a heterogeneous system and the formation of oil enriched mixed micelles [11]. In addition to its role on the lipid digestion, we have recently shown that the I-PC may have an important effect on the interaction of edible lipid nanocarriers with the intestinal mucus [3].

The therapeutic effect of oral nanocarriers depends on the fraction of formulations that reaches the intestinal epithelium. Then, it is advisable to use experimental techniques like PT for characterization, as it provides reliable information about the mucodiffusion capacity of the different populations that constitute the formulation after their interaction with the intestinal milieu.

As shown above, if RSS→TSS the use of *R*^2^ is clearly limited as the single screening parameter in PT analysis. This mathematical issue complicates the accurate analysis of the diffusion of the different populations Briefly, *RSS* gives an indication of the discrepancy between a theoretical model ($MSD=4D\pi \tau^{\alpha }$ in our case) and the experimental data. Interestingly, *RSS* value is proportional to the magnitude of the observable, reaching high values when MSD=MSD(τ) and a reduced magnitude for low and ~ constant *MSD* values (typical behavior of mucoadhesive formulations). Low *RSS* values of mucus retained particles make this parameter an interesting candidate as additional screening criterion to determine the goodness of fit; allowing the discrimination between non-diffusing particles and experimental issues. An alternative to *RSS* is the use of the root mean square Error (*RMSE*), which weights *RSS* and presents the same units as *MSD* (detailed information of the calculation of *RSS* and *RMSE* can be found in S.1 in Supplementary Information).

Bearing this in mind, we have analyzed the effect of the inclusion of *RSS* as an additional screening parameter on the determination of the mucodiffusion at two different conditions: (i) a non-digested lipid nanocarrier and (ii) the same nanocarrier pre-incubated in simulated intestinal fluid (which resulted on the formation of a heterogeneous system coated by intestinal enzymes and bile salts) [3,43,44]. First, we analyzed the effect of the *R*^2^ threshold on *α*, *D_m_*/*D_w_*, and the fraction of the formulation included in each *R*^2^ box (See Table 1 and Table 2). In both conditions, the classical approach (considering only the trajectories with *R*^2^ ≥ 0.6, 0.7, …) results in a loss of ~60–70% of the raw experimental data. Additionally, as observed with the model PS_NPs_, the digested formulation could be considered as highly-, partially-, or even non-retained in the mucus barrier depending on the goodness-of-fit threshold. *D_m_*/*D_w_* vs. *R*^2^ dependence was slightly lower for the mucoadhesive non-digested lipid nanocarrier; however, even for this formulation, *α* values increased from 0.22 to 0.78 as a function of the *R*^2^ threshold.

**Figure 5:**
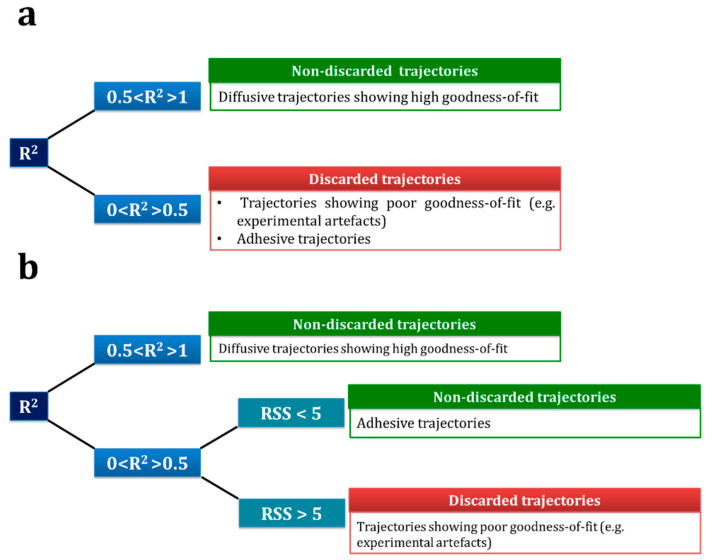
Optimized decision-tree for particle tracking (PT) screening of trajectories showing poor goodness-of-fit. (**a**) Conventional approach, based on $R^{2}$ and (**b**) proposed approach, based on a combination of $R^{2}$ and residuals sum of squares (RSS) error measurements.

**Figure 6:**
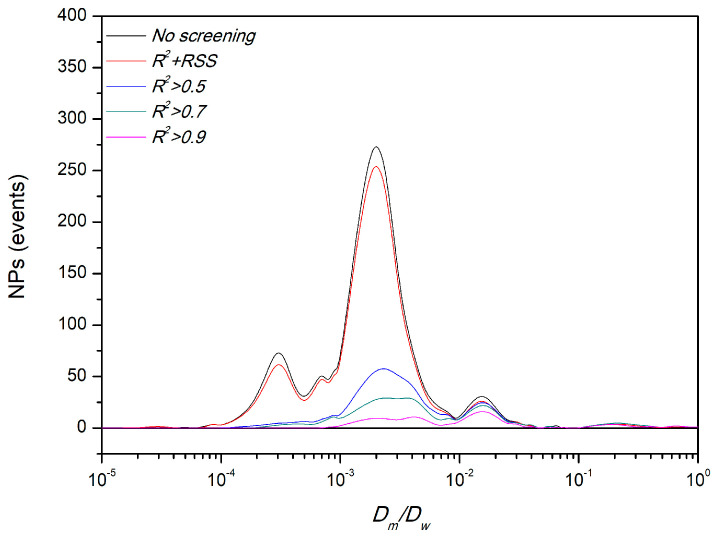
Distribution of *D_m_*/*D_w_* results of non-digested lipid nanocarrier after following different goodness-of-fit screenings.

To get a better insight of the effect of the use of *R*^2^ threshold or a combination of *R*^2^ *+ RSS* as a screening parameter, we followed the decision tree depicted in Figure 5. The mean results of the application of the combination approach to the non- and digested lipid nanocarriers are also included in Table 1 and Table 2. Figure 6 illustrates the results of both situations for the non-digested nanocarrier, which shows that the combination approach (*R*^2^ *+ RSS*) led to a qualitatively identical density line that the non-screened data (including >95% of the original trajectories). This clearly indicates a homogeneous distribution of the erratic trajectories (just ~4% of the original raw data). In line with our previous results, when the *R*^2^ threshold was used as a unique criterion to evaluate the goodness-of-fit, a clear reduction in the number of the slower trajectories was observed (the digested formulation presented a similar behavior, see Figure S1). This would lead to a clear overestimation of the final dose that may reach the intestinal epithelium.

**Figure 7:**
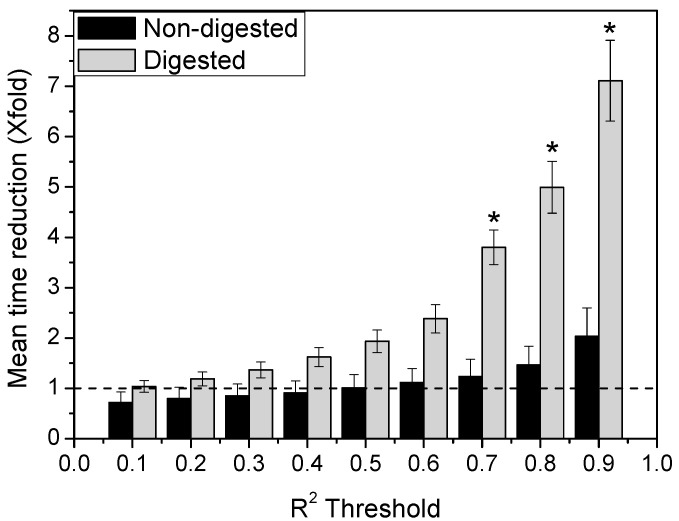
Reduction on the mean time required by non-digested (black columns) and digested (grey columns) lipid nanocarriers to diffuse across an intestinal porcine mucus layer of 100 µm based on a single (*R*^2^) or combination (*R*^2^ + RSS) screening approach (dotted line) (* *p* < 0.05).

Bearing this in mind, we have estimated the kinetic for the passage of both lipid nanocarriers (non-digested or digested) across the intestinal mucus. Considering that the intestinal mucus blanket has a mean thickness of ~100 μm, it is possible to use $\hat{D_{m}}$ to calculate the mean time required for a formulation to overcome this layer as (Equation (5):

(5) $$r=\sqrt{\hat{D_{m\;}}tlog\left(1/1-p\right)},$$

where *r* is the distance (100 μm), t is the time, and p the probability (set as 99.9%) [11,50,51]. Figure 7 shows the reduction on the mean time required for both lipid nanocarriers to diffuse across an intestinal porcine mucus layer of 100 μm based on a single (*R*^2^) or combination (*R*^2^ *+ RSS*) screening approach. Depending on the lipid nanocarrier, i.e., digested or non-digested, the single approach led up to ~2–8 fold reduction of the estimated diffusion mean time in comparison with the combination approach. These results clearly show that the *R*^2^ trim may result in an overestimation of D. Therefore, the fraction of particles that may reach the epithelium and then could be absorbed, would also be overestimated. This discrepancy between the estimation and reality could lead to misleading predictions regarding the expected oral pharmacokinetic profile of the nanocarrier.

**Figure 8:**
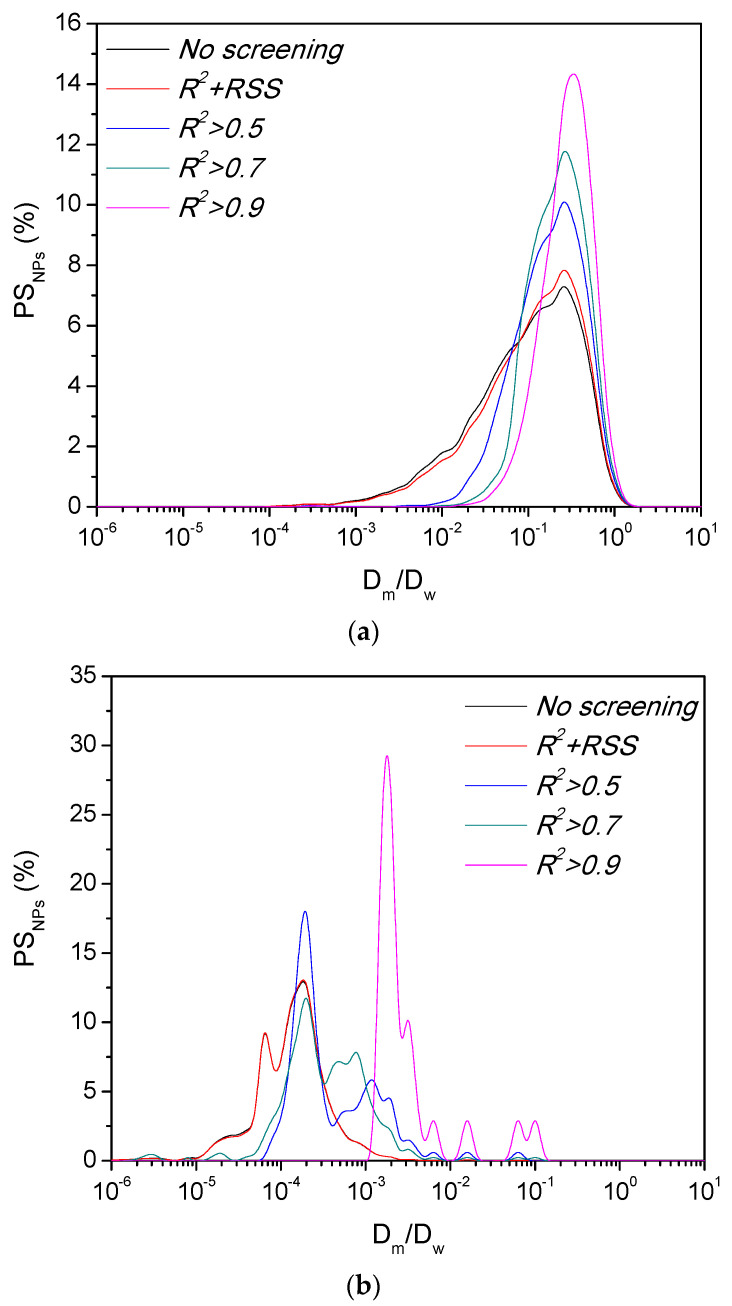
Particle populations considered to calculate the diffusion capacity of (**a**) diffusive PS_NPS_ control and (**b**) adhesive PS_NPS_ control after following different approaches to evaluate the goodness-of-fit.

To confirm the reliability of the combination approach, we followed the same decision tree illustrated in Figure 5 for the PS_NPs_ controls. Figure 8 shows the different particle populations considered for calculating the diffusion capacity of each PS_NPs_ control as a function of the screening approach. These results are in line with the ones described in Section 3.2 as well as those results obtained with the lipid nanocarriers. Interestingly, the application of the combination approach yielded a *D_m_*/*D_w_* ~ 1.7 × 10^−1^ (*α* ≈ 0.7) for the mucodiffusive PS_NPs_, a similar value to the one obtained using just *R*^2^ as screening parameter. This supports our hypothesis about the reliability of the use of *R*^2^ as single screening parameter for diffusing particles; meanwhile a *D_m_*/*D_w_* ~ 1.3 × 10^−3^ (*α* ≈ 0.1) was obtained for the mucoadhesive PS_NPs_ by using the combination approach. Both results agree with the behavior expected for this type of system [25,37,52].

These results show that the combination approach is the most reliable to properly describe the diffusion pattern of the lipid nanocarrier in porcine intestinal mucus. Then, another issue to determine which statistical measurement, mean, or median is the best estimator to express the results. Although mean value is the most used statistical measurement, density lines from Figure 6, Figure S1 (lipid nanocarriers), and Figure 8 (PS_NPs_) clearly show some non-gaussian distributions, indicating that the use of the median instead of the mean value is more precise in those cases [42]. Following this rationale, we calculated the mean/median ratio for each one of the formulations after the screening of the raw data with the combination approach (*R*^2^ *+ RSS*). Lipid nanocarriers displayed a mean/median ratio of 6.4 (digested) and 8.4 (non-digested) where the PS_PNs_ presented a ratio of 10.3 (mucoadhesive control) and 1.5 (mucodiffusive control). These results are in line with the *D_m_/D_w_* distributions from Figure 6, Figure S1 (lipid nanocarriers), and Figure 8 (PS_NPs_). For those formulations with a non-gaussian *D_m_/D_w_* distribution (both lipid nanocarriers and the mucoadhesive PS_NPs_), using the mean value to express the mucodiffusion capacity, instead of the median, may lead to an overestimation of one order magnitude of the results. However, for formulations with a homogeneous behavior (gaussian) in the intestinal mucus, like the mucodiffusive PS_NPs_ control, using the mean or the median value for determining its mucodiffusion capacity is not that relevant.

Altogether, this work shows that it is crucial to follow a proper statistical analysis of the original raw data in PT to extract accurate and reliable information about the interaction of the different particle populations in a heterogeneous formulation. This aspect is especially relevant to correctly determine the fraction of the formulation that would reach the intestinal epithelium after oral administration. Briefly, the use of just *R*^2^ to determine the goodness-of-fit of the raw trajectories to the $MSD=4D\tau^{\alpha }$ theoretical model may lead to the consideration of mucoadhesive particles as outliers and be discriminated for the estimation of the mucodiffusion of the whole formulation. Additionally, the calculation of the mean/median ratio of the results will illustrate the formulation heterogeneity. In this case, homogeneous (gaussian) formulations will have mean/median values ~1 and the heterogenous formulations mean/median values ≠1, indicating that the use of the mean instead of the median would lead to misleading results.

PT is a powerful technique for analyzing the capacity of nanocarriers to overcome the intestinal mucus barrier, but this great potential can be compromised if the proper statistical tools to screen and express the experimental results obtained in the laboratory are not correctly applied.

### 3.4. PT Software Implementation

The benefit of using PT for particle characterization compared to dynamic light scattering or FRAP is its capacity to define the behavior of each single particle of the sample, as observed for lipid nanocarriers [6,7,14,34]. This allows the classification of the particles within subpopulations providing information about the heterogeneity and polydispersity of the sample [6,9,14,53,54]. For this purpose, it is necessary to:

(I). Select an appropriate lag time threshold.
(II). Accurately screen the goodness-of-fit of the experimental data to the mathematical model.
(III). Perform an individual analysis of each trajectory.
(IV). Group similar trajectories into subpopulations.
(V). Express results in an easy-comprehensive fashion, including *D* and *α*, as well as other parameters that can help to understand the interaction of the nanocarrier with the intestinal mucus.

These are labor-intensive and complicated parts of PT experiments, since they entail the accurate processing of large sets of data.

To the best of our knowledge, there is no PT software available for screening trajectories based on additional error measurements to *R*^2^. Considering the relevance of the results previously shown, we have developed a software that integrates this screening approach and quickly analyses PT raw data, being an intuitive and simple software, that requires no previous programming skills.

The final application can be found at https://shiny.uclm.es/apps/tracking/ (accessed on 15 January 2021), where an account can be requested to the manuscript authors to get access. The organization in different tabs allows researchers to simulate, visualize, and analyze PT data.

Figure S2 shows the “Model” tab of the application, where the user can explore the theoretical model in Equation (1) for different values of D and *α*, and for different lag times. Two separate models can be represented to compare their theoretical shape for the next steps. Furthermore, in the “Simulation” tab (Figure S3), the behavior of two particles can be simulated for each model, including different levels of noise and the time lags at which the particles would stop diffusing. By setting that maximum lag time, users can visualize what can be expected to see in an experimental cloud of points. The simulated data can be downloaded for further use.

In the “Tracks” tab, the web application helps in the lag time decision step by visualizing the observed tracks. An Excel file with the raw *MSD* vs. time data can be uploaded. In addition to the data sheet, a set of metadata must be included in a separate sheet within the same spreadsheet file (including sample name, *D_w_*, etc., …). Once the file is loaded, the metadata are displayed, and a selectable table shows a list of the videos in the file on the right side. When a video is selected, the main pane of the application shows a table with the list of particles within that video, including their ID (*j* in the notation used above), the number of segments (*n_ij_* in the notation), and maximum *MSD* reached by that particle. The table is sortable and paged so as users can explore them. The video of the prototype particle is automatically computed and plotted, as a particle whose *MSD* at each lag time τ is the median of all the particles *MSD* at lag time *τ*, i.e., $MSD_{i0k}=median_{j}(MSD_{ijk})$. In addition to the prototype particle, further particles can be represented by just selecting them from the table. Figure S4 shows tracks from a given video and its prototype particle (0, the red one). This can be done for any number of particles, in any of the videos in the file, until the researcher decides the appropriate lag time. A slider on the right pane can be used for stretching the x scale of the chart and check different lag times. Moreover, the regression line for each represented trajectory can be shown by switching the “Show fitted line(s)” switcher. Note that when selecting the “Linear” tab of the representation, the linear transformation in Equation (3) is represented and fitted, instead of the original data. Figures S4 and S5 show the particles with their non-linear and linear regression fitted curves at the maximum lag time set to 1 s, respectively.

After exploring the tracks and decide on the lag time, we can go to the “Model” fitting tab. The “Fit models” button starts a process for fitting the models for all the particles in the file, or just the ones in the “Tracks” tab selected video. The non-linear fitting or the log-transformed linear fitting can be selected with the “Fit type” switch. Then, the following estimates, and their counterpart diagnostic measures, are shown in a table, see Figure S6: Video and particles IDs; Number of segments; α* (max (0, *α*); *D*; *D_short_* (*D* computed at the desired “Short lag time”); *D*/*D_w_*; *R*^2^; *RSS*; RMSE. The table is paged and sortable, so particle estimations and measurements can be easily explored.

The screening process is done afterwards by setting the screening filters, i.e., (i) the minimum value of *R*^2^; (ii) the maximum value of RSS (e.g., *R*^2^ ≥ 0.5; RSS ≤ 5, as proposed in the decision tree in Figure 5); and (iii) the minimum/maximum value of *α* (α*). It is important to highlight that even though the default values in Figure S6 are initially set, they can be changed at the analyst criteria to handle the problem at hand. The data included in the table can be downloaded in csv and xlsx formats for further study. The information included below the fittings table, e.g., summary statistics and histograms, are shown for the estimates and for the diagnostics measures, see Figure S7. The histograms provide meaningful information about the heterogeneity behavior of the sample, facilitating the decision about the statistical measurement (namely the mean or the median) to be selected for the final interpretation of results.

Once the screening and analysis step is finished, the overall diffusion parameters can be immediately seen in the “Model selection” tab (see Figure S8). The mean or the median can be chosen as summary statistic for the coefficients. The final model is represented in a plot, and the selected estimates and diagnostic measures are shown. Additionally, *α* values appear grouped in the different populations described in Section 2.6.2.

Besides, the software also includes a “Team” tab, which gathers the contact information of the software and methodology developers, as well as a “Support” tab, in which a summary of basic software instructions and a video tutorial (see S.2 in Supplementary Material) can be found.

Further research paths will be to investigate the properties of the so-called “prototype” particle and the use of non-linear profiles as a complementary screening method, as described in Cano et al. [49]. Moreover, data mining techniques such as unsupervised classification (cluster analysis) will be explored for subpopulations detection, as well as machine learning techniques for anomaly detection in the screening phase.

## 4. Conclusions

PT is a powerful and unique technique for studying the behavior of individual particles in biological conditions; in particular, this work focuses on oral drug delivery. This great potential has been exploited thanks to the development of high-speed and sensitivity cameras. However, once the experimental data have been obtained, there is a lack of consensus about their statistical analysis. Selecting the proper analysis would avoid overestimating the therapeutic potential of nanocarriers and achieving erroneous correlations between the physicochemical properties of the nanocarriers and their in vitro/in vivo performance. Both aspects are crucial for the design and development of new oral drug delivery formulations.

In this sense, this work proposes the use of a combination of parameters to correctly determine the goodness-of-fit of the experimental data to the mathematical diffusion model. We also consider the use of the mean/median ratio as a clear indicator of the heterogeneity of the formulation behavior in biological fluids. Finally, all these statistical aspects have been packaged in free, time saving, and user-friendly software to facilitate the essential task of the statistical analysis of raw PT data.

## Figures and Tables

**Table 1:** Main results of particle tracking experiments performed for digested lipid nanocarriers obtained after following different approaches for the screening of trajectories showing poor goodness-of-fit.

Screening Approach	*α*	*D_m_*/*D_w_*	NPs	NPs (%)
*R*^2^ ≥ 0.0	0.37	0.064	6965	100.0
*R*^2^ ≥ 0.1	0.47	0.083	5259	75.5
*R*^2^ ≥ 0.2	0.52	0.094	4532	65.1
*R*^2^ ≥ 0.3	0.57	0.108	3883	55.8
*R*^2^ ≥ 0.4	0.62	0.126	3236	46.5
*R*^2^ ≥ 0.5	0.66	0.148	2646	38.0
*R*^2^ ≥ 0.6	0.71	0.181	2090	30.0
*R*^2^ ≥ 0.7	0.76	0.225	1591	22.8
*R*^2^ ≥ 0.8	0.83	0.299	1102	15.8
*R*^2^ ≥ 0.9	0.92	0.443	627	9.0
*R*^2^ + *RSS*	0.32	0.064	6681	95.9

**Table 2:** Main results of particle tracking experiments performed for non-digested lipid nanocarriers obtained after following different approaches for the screening of trajectories showing poor goodness-of-fit.

Screening Approach	*α*	*D_m_*/*D_w_*	NPs	NPs (%)
*R*^2^ ≥ 0.0	0.22	0.009	1167	100.0
*R*^2^ ≥ 0.1	0.35	0.014	741	63.5
*R*^2^ ≥ 0.2	0.41	0.017	605	51.8
*R*^2^ ≥ 0.3	0.46	0.020	517	44.3
*R*^2^ ≥ 0.4	0.50	0.023	449	38.5
*R*^2^ ≥ 0.5	0.53	0.025	396	33.9
*R*^2^ ≥ 0.6	0.56	0.029	342	29.3
*R*^2^ ≥ 0.7	0.59	0.035	282	24.2
*R*^2^ ≥ 0.8	0.66	0.044	211	18.1
*R*^2^ ≥ 0.9	0.78	0.072	122	10.5
*R*^2^ + *RSS*	0.22	0.009	1113	95.4

## Data Availability

Data is contained within the article or Supplementary Material.

## Supplementary Materials

The following are available online at https://www.mdpi.com/1999-4923/13/3/370/s1; Figure S1: Distribution of *D_m_*/*D_w_* results of digested lipid nanocarrier after following different goodness-of-fit screenings; Figure S2: PT software. Model tab. Mathematical model representation; Figure S3: PT software. Simulation tab; Figure S4: PT software. Tracks tab. Non-linear regression of selected particles from the loaded data (mucodiffusive PS_NPs_ model); Figure S5: PT software. Tracks tab. Linear regression of selected particles from the loaded data; Figure S6: PT software. Model fitting tab. Videos and fitting type (linear or non-linear) can be selected; Figure S7: PT software. Model fitting tab. Distribution of obtained estimators; Figure S8: PT software. Model selection tab. Overall parameter estimates and diagnostic measures.
