# Supplementary material for: Taking Particle Tracking into Practice by Novel Software and Screening Approach: Case-Study of Oral Lipid Nanocarriers
Source: Pharmaceutics. 2021 Mar 10;13(3):370. doi: 10.3390/pharmaceutics13030370 (PMC8001040; doi:10.3390/pharmaceutics13030370)
Supplement: Supplementary file 1 [file pharmaceutics-13-00370-s001.pdf]

pharmaceutics

MDPI

# Supplementary Materials: Taking Particle Tracking into Practice by Novel Software and Screening Approach: Case-Study of Oral Lipid Nanocarriers

María Plaza-Oliver, Emilio L. Cano, María Mar Arroyo-Jimenez, Matías Gámez, María Victoria Lozano-López and Manuel J. Santander-Ortega

## S.1. Model assessment

After fitting one particle's model, either from the nonlinear model or from the linear model, the predicted MSDs are computed using the parameter estimates $\widehat{D}_{ij}$ and $\widehat{\alpha}_{ij}$ (Equation (S1)):

$$\widehat{MSD}_{ijk} = 4\widehat{D}_{ij}\tau_k^{\widehat{\alpha}_{ij}} \quad (S1)$$

that are subtracted from the observed $MSD_{ijk}$ (Equation (S2)) to get the residuals of particle i in video j, $e_{ijk}$:

$$e_{ijk} = MSD_{ijk} - \widehat{MSD}_{ijk} \quad (S2)$$

The following measures for the model adequacy are then computed for each trajectory: i) the Residuals Sum of Squares (RSS), Equation (S3); ii) the Root Mean Square Error (RMSE), Equation (S4); and the coefficient of determination $R^2$, Equation (2).

$$RSS_{ij} = \sum_k e_{ijk}^2 \quad (S3)$$

$$RMSE_{ij} = \sqrt{\frac{\sum_k e^2{}_{ijk}}{n_{ij}}} \quad (S4)$$

Care should be taken when computing $R^2$ goodness-of-fit measure, as the statistical software usually provides linear determination coefficient $R^2$ for the regression line in Equation (3), which is close, but not equal, to the coefficient of determination $R^2$ of the estimated model in Equation (1).

## S.2. Basic instructions for the use of developed particle tracking software

1. Enter the app and log in with your supplied credentials:

https://shiny.uclm.es/apps/tracking/

2. Prepare your raw data (excel with MSDs) according to the requirements of the app. There are some examples loaded that can be downloaded and used as templates in this process:

Tracks→file sources→example files→select the file (i.e. diffusive)→download

In further detail, the excel file must contain two sheets:

Sheet 1 must be named Tracks MSD and must include the specific file and column names and information that appear in the example file. The user must replace specific information (i.e. concerning MSD or Number of segments) by his/her own raw data.

Sheet 2 must be named config and must include all the information that includes the example file (i.e. Dt in msec).

3. Once the excel file is prepared, it must be loaded in the app:

Tracks→file sources→upload file→select the file (excel in the format detailed in point 2 containing the user's raw MSDs)→ load tracks

4. Select the desired video. All the particles in those videos will appear in a table showing their máx. MSDs. Any particle can be selected and it will appear in the upper graph, which also shows the median of all the particles in that video (prototype video).
5. Select lag time in its scroll bar.
6. Enter model fitting tab.
7. Select which video the user desires to analyse, the fitting approach (lineal, non-lineal), and the screening parameters. α values corresponding to superdiffusive particles (higher than 1.1) can also been excluded. An additional lag time can be selected.
8. Select fit model.
9. Results will appear in a table. The statistics behavior can be seen at a glance by several histograms.
10. Model selection tab shows a summary of the results.

Please, do not hesitate to contact us with any comment or doubt. A video tutorial can be found at: https://shiny.uclm.es/apps/tracking/

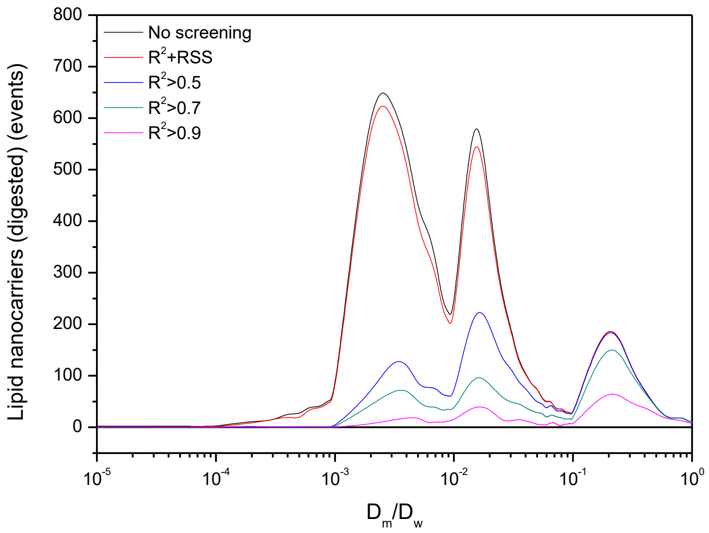


**Figure S1.** Distribution of $D_m/D_w$ results of digested lipid nanocarrier after following different goodness-of-fit screenings.

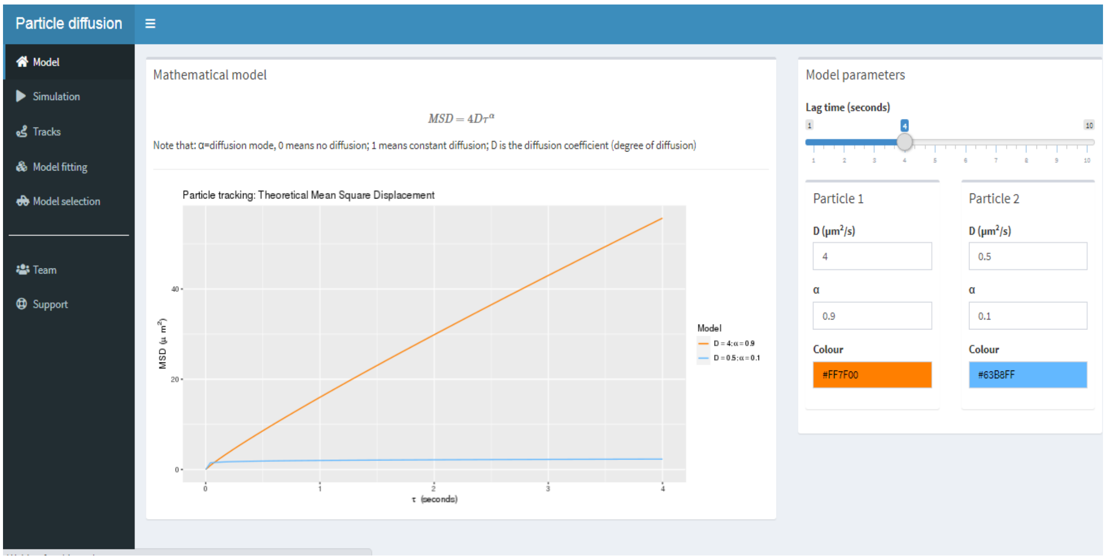


**Figure S2.** PT software. Model tab. Mathematical model representation. Lag time, D, α and colour can be modified for any of the particles.

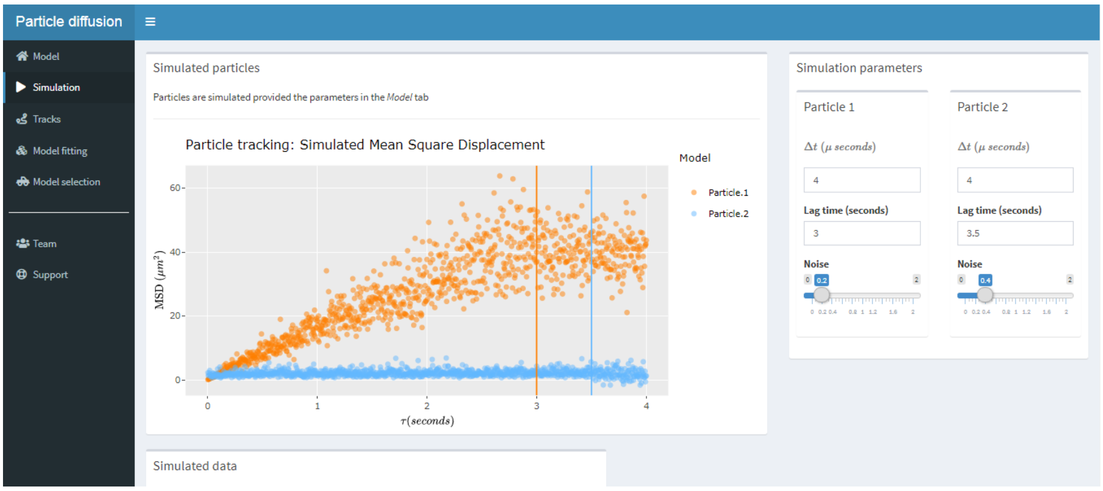


**Figure S3.** PT software. Simulation tab. Noise, lag time and Δt can be adjusted for any of the simulated particles.

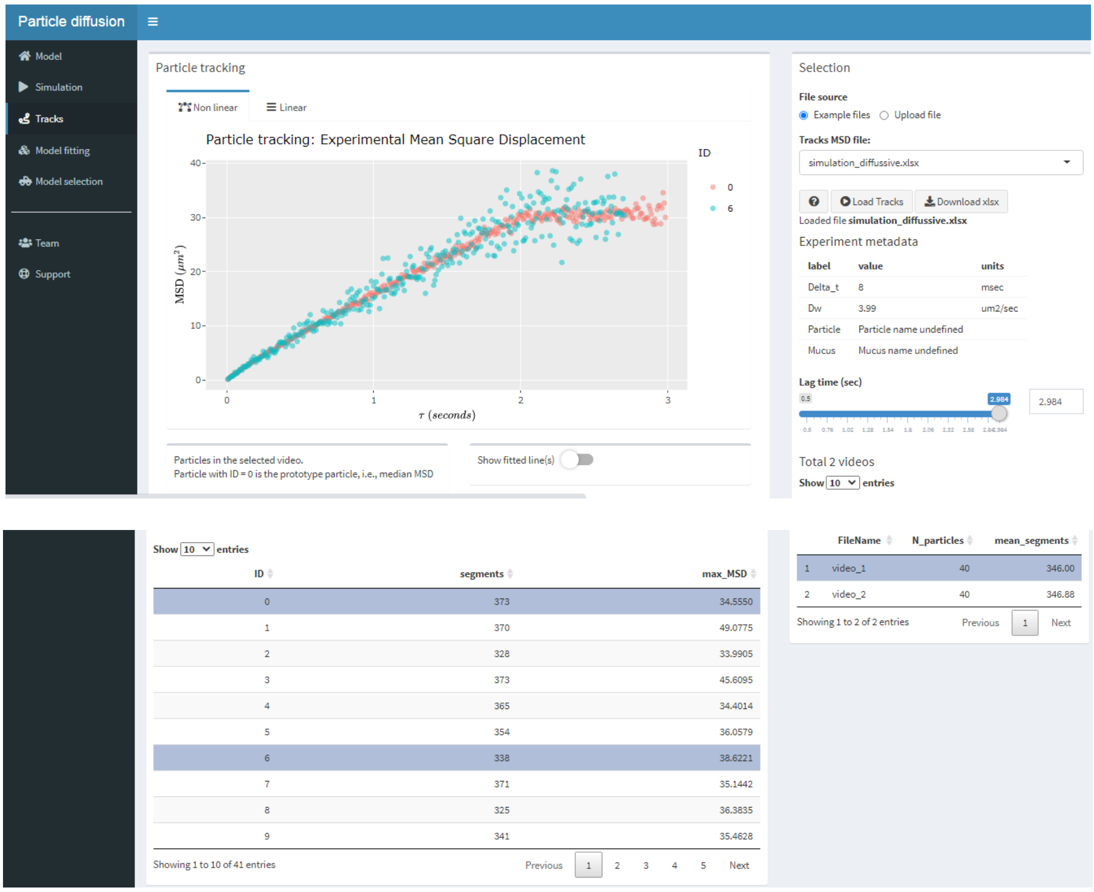


**Figure S4.** PT software. Tracks tab. Non-linear regression of selected particles from the loaded data (mucodiffusive $PS_{NPs}$ model). Examples files are included. Lag time can be adjusted. The prototype particle is shown in red (readers are referred to the online version of the paper).

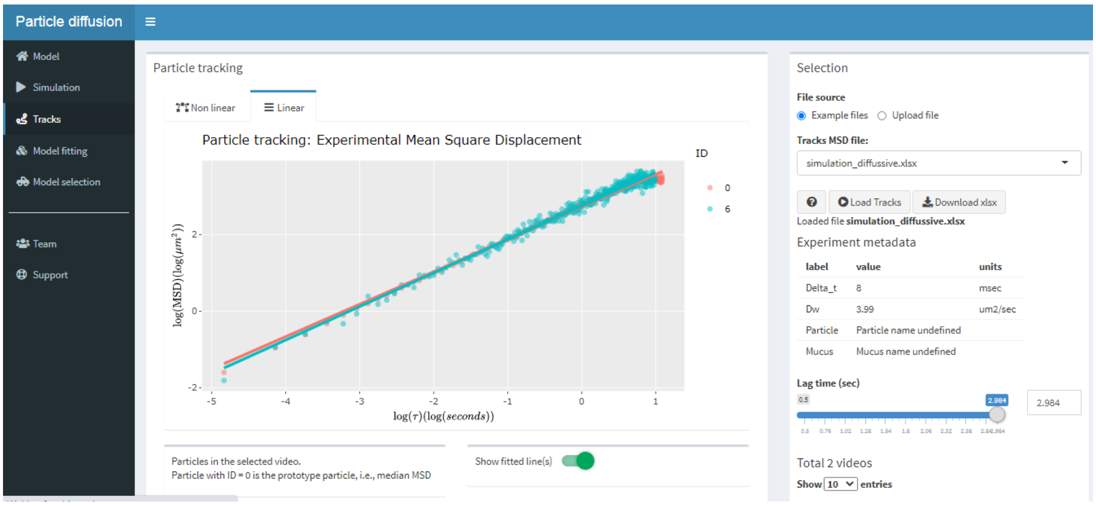


**Figure S5.** PT software. Tracks tab. Linear regression of selected particles from the loaded data. Examples files are included. Lag time can be adjusted. The prototype particle is shown in red (readers are referred to the online version of the paper).

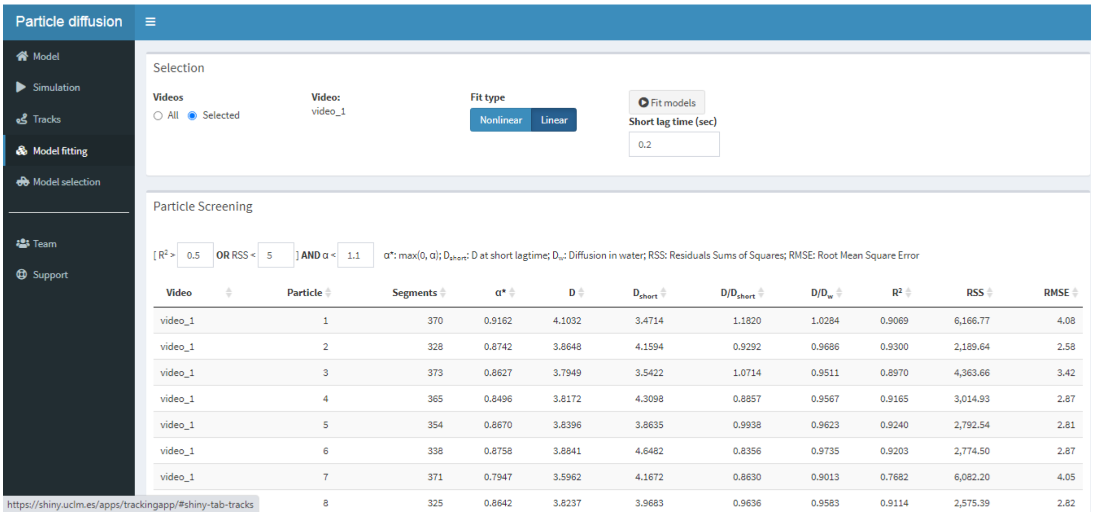


**Figure S6.** PT software. Model fitting tab. Videos and fitting type (linear or non-linear) can be selected. A base lag time can be included, in order to obtain $D_{long}/D_{short}$. Screening parameters, i.e. $R^2$, RSS and α can be selected. The table shows estimates of several parameters related to diffusion, such as D, $D/D_w$, $D_{long}/D_{short}$ or α, as well as error estimators, like $R^2$, RSS or RMSE. Data can be exported to an excel file if desired.

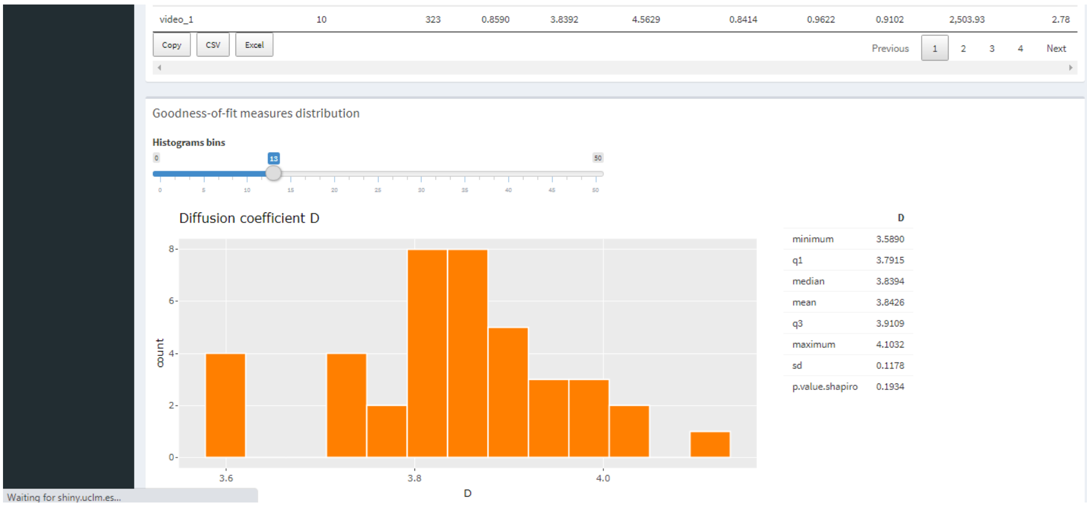


**Figure S7.** PT software. Model fitting tab. Distribution of obtained estimators, e.g. D or α. The number of histograms bins can be modulated.

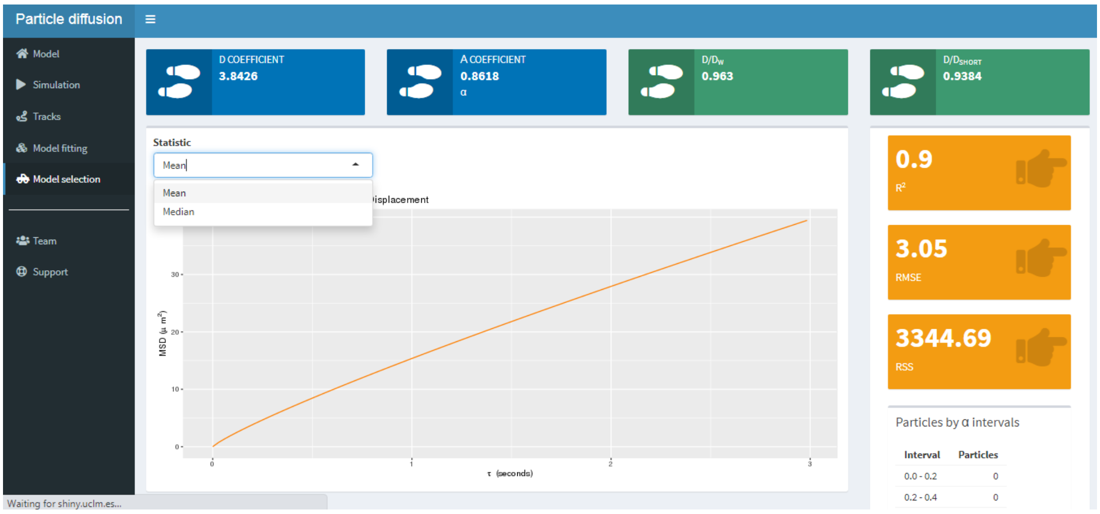


**Figure S8.** PT software. Model selection tab. Overall parameter estimates and diagnostic measures. It includes a classification of particles depending on their α values.
